# Systematic review of the appropriateness of eye care delivery in eye care practice

**DOI:** 10.1186/s12913-019-4493-3

**Published:** 2019-09-06

**Authors:** Kam Chun Ho, Fiona Stapleton, Louise Wiles, Peter Hibbert, Sally Alkhawajah, Andrew White, Isabelle Jalbert

**Affiliations:** 1 School of Optometry and Vision Science, UNSW Sydney, Sydney, NSW 2052 Australia; 2 Eye Health, Injury Division, The George Institute for Global Health, Sydney, Australia; 3 Faculty of Medicine and Health Sciences, Australian Institute of Health Innovation, Macquarie University, Sydney, New South Wales Australia; 4 Centre for Population Health Research, School of Health Sciences, University of South Australia, Adelaide, South Australia Australia; 5 Optometry and Vision Science Department, King Saud University, Riyadh, Saudi Arabia; 6 Save Sight Institute, University of Sydney, Westmead Hospital, Sydney, New South Wales Australia; 7 Centre for Vision Research, Westmead Institute for Medical Research, University of Sydney, Westmead Hospital, Sydney, New South Wales Australia

**Keywords:** Glaucoma, Delivery of health care, Diabetic retinopathy, Public health, Evidence-based practice, Process assessment (health care)

## Abstract

**Background:**

Health care systems are continually being reformed, however care improvement and intervention effectiveness are often assumed, not measured. This paper aimed to review findings from published studies about the appropriateness of eye care delivery, using existing published evidence and/or experts’ practice and to describe the methods used to measure appropriateness of eye care.

**Methods:**

A systematic search was conducted using Medline, Embase and CINAHL (2006 to September 2016). Studies reporting the processes of eye care delivery against existing published evidence and/or experts’ practice were selected. Data was extracted from published reports and the methodological quality using a modified critical appraisal tool. The primary outcomes were percentage of appropriateness of eye care delivery. This study was registered with PROSPERO, reference CRD42016049974.

**Results:**

Fifty-seven studies were included. Most studies assessed glaucoma and diabetic retinopathy and the overall methodological quality for most studies was moderate. The ranges of appropriateness of care delivery were 2–100% for glaucoma, 0–100% for diabetic retinopathy and 0–100% for other miscellaneous conditions. Published studies assessed a single ocular condition, a sample from a single centre or a single domain of care, but no study has attempted to measure the overall appropriateness of eye care delivery.

**Conclusions:**

These findings indicated a wide range of appropriateness of eye care delivery, for glaucoma and diabetic eye care. Future research would benefit from a comprehensive approach where appropriateness of eye care is measured across multiple conditions with a single methodology, to guide priorities within eye care delivery and monitor quality improvement initiatives.

**Electronic supplementary material:**

The online version of this article (10.1186/s12913-019-4493-3) contains supplementary material, which is available to authorized users.

## Background

Globally, 285 million people of all ages suffer from visual impairment [1]. Long-term ocular conditions, including both ocular diseases (e.g. glaucoma, diabetic retinopathy, age-related macular degeneration and cataract) and uncorrected refractive errors are the major causes of visual impairment worldwide [2]. The prevalence of vision problems is strongly associated with ageing and this compromised visual function affects individuals’ ability to perform activities of daily living [3]. Common eye diseases can often be detected early and their visual impact minimised or they can be prevented by appropriate eye care services, including routine eye examinations [4–6]. Due to the growing demand for eye care in the context of resource scarcity, interest in measuring and improving the appropriateness of eye care delivery is growing [7, 8]. Appropriate care is defined as provision of evidence-based care that is relevant to the patient’s needs and based on established standards [9].

Translation of best available evidence into clinical practice is important, ensuring that both efficacy and cost-effectiveness of patient management is maintained [10]. Evidence-based guidelines aim to translate well conducted scientific trials into easy to apply recommendations. Such guidelines intend to guide practitioners and help them to improve their professional practice and optimize patient care [11]. Evidence-based guidelines are not always adhered to and/or fully implemented in the clinical setting. Adherence to guidelines can be quantitatively measured using quality indicators of appropriateness of care delivery. Quality Indicators can be defined as “measurable components of a standard or guideline, with explicit criteria for inclusion, exclusion, time frame, setting and compliance action” [12].

Evidence of suboptimal care being delivered exist, arising from several large studies assessing appropriateness of care across different health conditions. The RAND study conducted in 2000 in the United States evaluated performance on 439 quality indicators of appropriateness of care for 30 acute and chronic conditions as well as preventive care. The RAND study showed that American adults received recommended care only 55% (range 11–79%) of the time [13]. More recently, the CareTrack study in Australia showed similar results with 57% (range 13–90%) of Australian adults receiving appropriate care across 22 conditions [12]. Ocular conditions were not included in the CareTrack study [12]. Defining existing eye care practice patterns and current variation from best practices is an important component of a systemic approach to improving appropriateness of eye care [14, 15].

## Purpose

This paper aimed to review findings from published studies about the appropriateness of eye care delivery, using existing published evidence and/or experts’ practice. A secondary aim was to describe and compare the variety of methods used to measure appropriateness of eye care.

## Methods

### Data sources and searches

A systematic search was conducted using Medline, Embase and the Cumulative Index to Nursing and Allied Health Literature (CINAHL) electronic databases to identify studies related to the appropriateness of eye care. The search strategy was reviewed and tested by an academic librarian and reviewed by content experts (IJ and FS). The literature review process followed the Preferred Reporting Items for Systematic reviews and Meta-Analyses (PRISMA) procedures [16] and the review protocol was published on PROSPERO (http://www.crd.york.ac.uk/prospero/, reference CRD42016049974). As eye conditions with higher prevalence and heavier burden on the health system, the emphasis was put on glaucoma, diabetic retinopathy, refractive error, cataract and macular degeneration [17]. The search incorporated the three elements:

1. Profession-specific terms: “Optometr*”, “Ophthalmolog*”, “General practitioner*”, “Orthopt*”, “Ophthalmic nurse*”, “Ophthalmic practitioner*”.
2. Subject headings: Exp”Quality of Health Care” in Medline, Exp”Health care quality” in Embase, MH”Health Services Research+” in CINAHL.
3. Condition-specific terms: Exp Glaucoma, Exp diabetic retinopathy, Exp refractive errors, Exp macular degeneration, Exp cataract.

An example of the full electronic search strategy for Medline database is illustrated in Additional file 1.

### Study selection

**Fig. 1:**
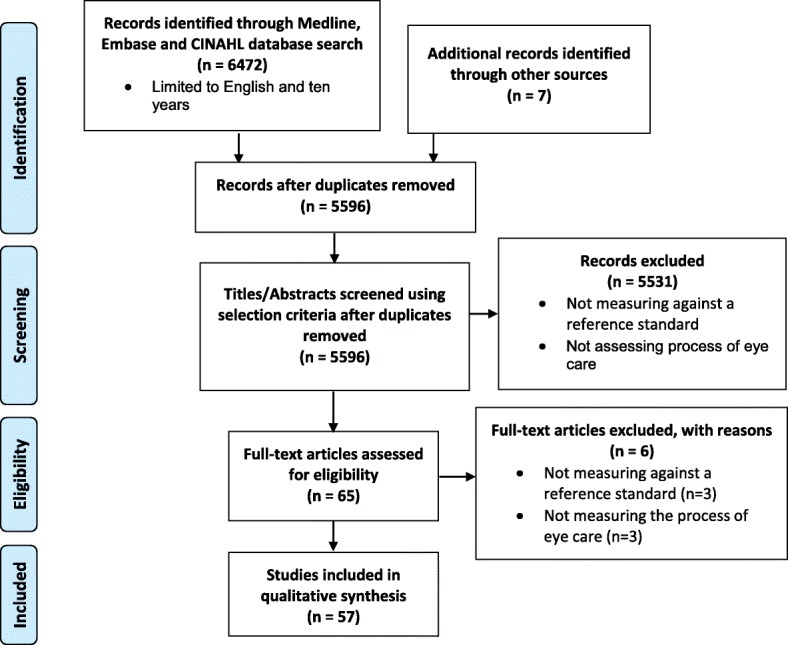
PRISMA flow diagram for appropriateness of eye care delivery

Reference lists and citations were used to cross-check the results of our search. The reference details and abstracts of the 5596 articles retrieved from the literature search after duplicates removal were reviewed by one reviewer (KCH). Studies assessing the processes of eye care delivery against existing published evidence and experts’ practice (e.g. consultant ophthalmologists’ practice) were included. Studies assessing outcomes of care delivery such as patient satisfaction or those assessing structural aspects of care delivery such as workforce characteristics, infrastructure, regulations and policies were excluded from analysis in this review. The search was not restricted by type of study design, and no other limitations (e.g. population, intervention, comparison, length of follow-up) were set. The search was limited to English and 10 years to the search date (2006 to 16th September 2016). Studies conducted more than 10 years ago were excluded, on the basis that appropriateness of care was likely to change over time, and that older studies might not reflect recent changes in care delivery standards [18]. The references were narrowed to 65 articles after title and abstract screening following the application of exclusion criteria (Fig. 1). A further six articles were excluded after full text review with three that did not access process of care and three that did not measure against existing published evidence or experts’ practice.

### Data extraction and quality assessment

Each paper was reviewed and information was extracted based on the following characteristics:

- Country
- Condition(s) – the eye condition(s) for which the appropriateness of care was assessed
- Professions – the health professions delivering the care of the assessed eye condition
- Methods – the method used to assess the appropriateness of eye care delivered
- Sample size
- Response rate
- Evidence sources – the reference standard used to assess the appropriateness of eye care delivered
- Settings – classification based on whether study was conducted in hospital or independent practice
- Number of sites – the number of sites that the study was conducted at
- Timing – the timing and visit types assessed in the article (e.g. at diagnosis, follow-up, etc)
- Percentage of encounters with appropriate eye care – the number of quality indicators met over the total number of relevant quality indicators

Taking into consideration the diversity of study types (e.g. descriptive, interventional and observational studies, record reviews, and surveys), two reviewers (KCH and SA) independently assessed the quality of each article using a validated critical appraisal tool [19]. The applied tool was modified by adding questions from other validated critical appraisal tools including Critical Appraisal Skills Programme (CASP) diagnostic checklist [20], National Institutes of Health (NIH) Quality Assessment Tool For Observational Cohort And Cross-Sectional Studies [21], Joanna Briggs Institute (JBI) Critical Appraisal Checklist For Studies Reporting Prevalence Data [22], Effective Public Health Practice Project (EPHPP) Quality assessment tool for quantitative studies [23].

The modified quality assessment tool included 17 individual criterions with questions from validated critical appraisal tools [20–23] (Additional file 2) and grouped in the seven categories listed below:

- Quality of reporting (adequate description of the context [19], clearly stated aims [19–21], eligibility [21], methods and findings [20])
- Selection bias (representative of the selected individuals [22, 23], response rate at least 50% [21], and sample size justification [21])
- Study design (presence of randomisation [23], presence of control group [19, 23])
- Blinding (blinding of outcome assessors to the intervention or exposure status of participants [20, 21, 23], blinding of participants to research question [23], and blinding of decision making between participants and experts [20])
- Data collection tools (reliability of the data collection tool [22, 23] and valid reference used to assess the appropriateness of care [20])
- Analysis (sufficient rigorous data analysis [19, 22, 23])
- Limitations (key potential confounders are identified and accounted for [21–23])

The number of criteria used varied depending on the study design of the publication being reviewed. An overall rating was allocated for each paper as a percentage based on the number of criteria met over the number of relevant criteria for the corresponding study design. If less than 60% criteria relevant to the study design was met, this item was scored as Weak in the quality assessment tool. It was scored moderate if 60–79% of criteria were met and strong if 80–100% of criteria were met. A third reviewer (IJ) resolved any disagreements and consensus was reached through discussion. All articles were included, and the results of critical appraisal are provided in Additional file 3.

### Data synthesis and analysis

Due to the anticipated heterogeneity of included studies, no plans were made to pool the results statistically, therefore a meta-analysis was not undertaken. For each study, the range of percentage of appropriate care (summary data from published reports, but not individual patient-level data) and the number of quality indicators were separated according to the nature of the quality indicators into the following six domains of care: ‘history taking’, ‘physical examination’, ‘management’, ‘recall period’, ‘referral’ and ‘patient education’. On occasion, data provided in the papers had to be reclassified to fit these proposed domains of care. Data were also reanalysed as required so that the results could be presented in terms of appropriateness to prescribed care and not the reverse (i.e. percentage with inappropriate care).

## Results

Of 6472 citations, 57 articles met the inclusion (see Fig. 1). The characteristics of these studies are presented in Table 1. The majority of the studies were from the United Kingdom (UK) (*n* = 25) and the United States of America (USA) (*n* = 15), with Australia (*n* = 5), Australia and New Zealand (NZ) (*n* = 2) and other countries accounting for the remainder. Among the 57 papers, two-thirds examined eye care delivery for glaucoma (*n* = 28) and diabetic retinopathy (*n* = 11). The majority of papers assessed the care delivered by optometrists (*n* = 22) and ophthalmologists (*n* = 19), with another seven studies including both professions. Half of the studies were rated moderate (60–79% of quality criteria met) for the methodological quality (*n* = 29), another one-third were rated strong (80–100% of quality criteria met) (*n* = 19) and the remainder were rated weak (< 60% of quality criteria met) (*n* = 9). For all conditions but diabetic retinopathy, a similar pattern of distribution of methodological quality (i.e. mostly moderate) was observed. However, for diabetic retinopathy most of the studies (73%) were rated strong in methodological quality.

**Table 1:** Study Characteristics (*n* = 57). USA = United States of America, UK=United Kingdom, NZ = New Zealand, A&E = accident and emergency

Country	Evidence sources	Year	Professions	Outcomes	Methods	Overall quality^a^	Author (reference)	n^b^
Glaucoma								
UK	Clinical practice guidelines [24, 25]	2013	Ophthalmologist	Current practice pattern	Record review	Strong	Fung et al. [26]	101
UK	Clinical practice guidelines [25]	2012	Ophthalmologist & optometrist	Guidelines adherence	Record review	Weak	Chawla et al. [27]	200
UK	Clinical practice guidelines [25, 28]	2012	Optometrist	Guidelines adherence	Record review	Moderate	Khan et al. [29]	114
UK	Clinical practice guidelines [30]	2012	Optometrist	Validation of self-reported practice	Interview with practitioner and unannounced standardised patient	Moderate	Theodossiades et al. [31]	34
UK	Clinical practice guidelines [25]	2011	Ophthalmologist	Current practice pattern	Practitioner Survey	Moderate	Stead et al. [32]	626 (69%)
UK	Clinical practice guidelines [33, 34]	2009	Optometrist	Quality of referral letter	Record review	Moderate	Scully et al. [35]	121
UK	Experts’ opinions	2012	Optometrist	Diagnostic accuracy	Clinical agreement with expert	Moderate	Marks et al. [36]	145
UK	Experts’ opinions	2011	Optometrist	Diagnostic accuracy	Record review	Moderate	Ho and Vernon [37]	140
UK	Experts’ opinions	2011	Optometrist	Quality of referral	Record review	Moderate	Shah and Murdoch [38]	110
UK	Experts’ opinions	2010	Optometrist	Feasibility of shared care	Record review	Strong	Syam et al. [39]	1184
UK	Experts’ opinions	2010	Optometrist	Quality of referral	Record review	Weak	Lockwood et al. [40]	441
UK	Experts’ opinions	2007	Ophthalmologist & optometrist	Diagnostic accuracy	Clinical agreement with expert	Strong	Azuara-Blanco et al. [41]	100
UK	Experts’ opinions	2006	Optometrist	Quality of referral	Record review	Weak	Patel et al. [42]	376
UK	Experts’ opinions	2006	Optometrist & associate specialists	Diagnostic accuracy	Clinical agreement with expert	Moderate	Banes et al. [43]	350
USA	Clinical practice guidelines [24, 44]	2016	Ophthalmologist	Current practice pattern	Record review	Moderate	Solano-Moncada et al. [45]	250
USA	Clinical practice guidelines [44]	2016	Ophthalmologist & optometrist	Current practice pattern	Claims data	Strong	Elam et al. [46]	56,675
USA	Clinical practice guidelines [47]	2015	Ophthalmologist	Diagnostic accuracy	Record review	Moderate	Zebardast et al. [48]	212
USA	Clinical practice guidelines [49] & experts’ opinions	2013	Ophthalmologist	Guidelines adherence	Record review	Strong	Ong et al. [50]	103
USA	Clinical practice guidelines [44]	2012	Ophthalmologist & optometrist	Current practice pattern	Claims data	Moderate	Swamy et al. [51]	143,374
USA	Clinical practice guidelines [49]	2007	Ophthalmologist	Guidelines adherence	Claims data, record review, practitioner survey and patient survey	Moderate	Quigley et al. [52]	300
USA	Clinical practice guidelines [53]	2006	Ophthalmologist	Current practice pattern	Claims data	Strong	Coleman et al. [54]	4427
Australia & NZ	Clinical practice guidelines [55]	2015	Optometrist	Current practice pattern	Practitioner Survey with case vignette	Moderate	Zangerl et al. [56]	818 (18%)
Australia & NZ	Clinical practice guidelines [47, 57, 58]	2008	Ophthalmologist	Current practice pattern	Practitioner Survey	Strong	Liu [59]	627 (78%)
Scotland	Clinical practice guidelines [25, 60]	2015	Optometrist	Quality of referral	Record review	Strong	El-Assal et al. [61]	1622
Scotland	Clinical practice guidelines [60]	2009	Optometrist	Quality of referral	Record review	Moderate	Ang et al. [62]	303
Canada	Clinical practice guidelines [63]	2014	Ophthalmologist & optometrist	Quality of referral letter	Record review	Moderate	Cheng et al. [64]	200
Germany	Clinical practice guidelines [57]	2008	Ophthalmologist	Guidelines adherence	Practitioner Survey	Moderate	Vorwerk et al. [65]	335 (12%)
Singapore	Clinical practice guidelines [66]	2008	Ophthalmologist	Current practice pattern	Practitioner Survey	Strong	Ang et al. [67]	126 (80%)
Diabetic retinopathy								
Australia	Clinical practice guidelines [68]	2011	Optometrist	Current practice pattern	Practitioner Survey	Weak	Slater and Chakman [69]	985 (26%)
Australia	Clinical practice guidelines [70]	2011	Optometrist	Current practice pattern	Practitioner Survey with case vignette	Strong	Ting et al. [71]	568 (57%)
Australia	Clinical practice guidelines [70]	2010	Ophthalmologist	Guidelines adherence	Practitioner Survey with case vignette	Strong	Yuen et al. [72]	480 (63%)
NZ	Clinical practice guidelines [73]	2012	Optometrist	Guidelines adherence	Record review	Strong	Hutchins et al. [74]	157
USA	Clinical practice guidelines [75]	2012	Ophthalmologist & optometrist	Current practice pattern	Patient survey	Strong	Chou et al. [76]	29,495
USA	Clinical practice guidelines [77]	2010	Ophthalmologist	Guidelines adherence	Record review	Strong	Tseng et al. [78]	70
Hong Kong	Clinical practice guidelines [79]	2016	General practitioner	Guidelines adherence	Practitioner Survey	Strong	Wong et al. [80]	414 (13%)
Bahrain	Clinical practice guidelines [81]	2014	General practitioner	Guidelines adherence	Record review	Strong	Al-Ubaidi et al. [82]	200
Switzerland	Clinical practice guidelines [83]	2013	General practitioner	Guidelines adherence	Record review	Moderate	Burgmann et al. [84]	275
UK	Clinical practice guidelines [85]	2011	General practitioner	Guidelines adherence	Record review	Strong	Mc Hugh et al. [86]	3010
Brazil	Clinical practice guidelines [87]	2007	General practitioner	Current practice pattern	Practitioner Survey	Weak	Preti et al. [88]	168 (34%)
Age-related macular degeneration								
Italy	Multiple clinical trials [89–92]	2016	Ophthalmologist	Guidelines adherence	Interview with patient	Moderate	Parodi et al. [93]	283
Turkey	Multiple clinical trials [89, 90, 94]	2015	Ophthalmologist	Current practice pattern	Practitioner Survey	Moderate	Muhammed et al. [95]	249 (21%)
UK	Multiple clinical trials [89, 96–99]	2013	Ophthalmologist & optometrist	Current practice pattern	Practitioner Survey with case vignette	Weak	Lawrenson and Evans [100]	1468 (15%)
USA	Multiple clinical trials [89, 101, 102]	2008	Ophthalmologist	Current practice pattern	Patient survey	Moderate	Charkoudian et al. [103]	332 (99%)
Cataract								
UK	Clinical practice guidelines [104]	2011	Ophthalmologist	Current practice pattern	Practitioner Survey	Weak	Gomaa and Liu [105]	158 (53%)
UK	Clinical practice guidelines [106]	2009	Optometrist & general practitioner	Quality of referral letter	Record review	Strong	Park et al. [107]	124
UK	Clinical practice guidelines [108]	2006	Optometrist	Quality of referral letter	Record review	Moderate	Lash et al. [109]	351
USA	Clinical practice guidelines [110]	2009	Resident ophthalmologist	Guidelines adherence	Record review	Strong	Niemiec et al. [111]	129
Preventative eye care								
UK	Clinical practice guidelines [112–114] & experts’ opinions	2009	Optometrist	Current practice pattern	Unannounced Standardised patient	Moderate	Shah et al. [115]	100
UK	Clinical practice guidelines [114, 116, 117] & experts’ opinions	2009	Optometrist	Current practice pattern	Unannounced Standardised patient	Moderate	Shah et al. [118]	102
UK	Clinical practice guidelines [114, 117, 119] & experts’ opinions	2008	Optometrist	Current practice pattern	Unannounced Standardised patient	Moderate	Shah et al. [120]	100
Australia	Multiple clinical trials’ results [89, 90, 121–128]	2015	Optometrist	Current practice pattern	Practitioner Survey	Moderate	Downie and Keller [129]	283 (6.7%)
Dry eye								
Australia	Clinical practice guidelines [130, 131]	2013	Optometrist	Guidelines adherence	Practitioner Survey	Moderate	Downie et al. [132]	144 (22%)
USA	Clinical practice guidelines [133]	2010	Ophthalmologist	Guidelines adherence	Record review	Weak	Lin et al. [134]	178
All ocular conditions at A&E								
UK	Experts’ opinions	2007	Optometrist	Diagnostic accuracy	Clinical agreement with expert	Moderate	Hau et al. [135]	150
Amblyopia								
USA	Multiple clinical trials [136, 137]	2013	Ophthalmologist	Guidelines adherence	Record review	Moderate	Jin et al. [138]	123
Esotropia								
USA	Clinical practice guidelines [139]	2010	Ophthalmologist	Guidelines adherence	Record review	Weak	Gupta et al. [140]	200
Non-infectious uveitis								
USA	Clinical practice guidelines [141]	2011	Ophthalmologist & rheumatologist	Current practice pattern	Record review and practitioner survey	Moderate	Nguyen et al. [142]	580

^a^If less than 60% criteria in the quality assessment tool were met, quality was scored as weak; it was scored moderate if 60–79% were met and strong if 80–100% were met. ^b^Response rate reported in bracket where applicable

**Fig. 2:**
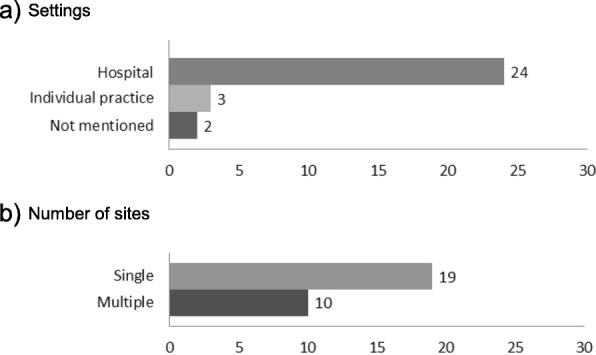
Review Site Characteristics (*n* = 29). The number on each bar indicates the number of included studies (**a**) assessed within the corresponding settings, (**b**) conducted at single or multiple sites. When eye care appropriateness was measured using record review, assessments were most frequently conducted at a single site (*n* = 19) and in these cases, studies were conducted in a hospital setting. Use of a single site reduces logistical challenges, but the results may not be generalisable to other environments with a different location, practice types and case-mix

Record review (26 of 57 studies) and practitioner survey with or without case vignettes (15 of 57 studies) were the most commonly used methods, with one study using a combination of both methods and one study using both methods with claims data and patient survey. When eye care appropriateness was measured using record review, assessments were most frequently conducted at a single site (*n* = 19) and in these cases, studies were conducted in a hospital setting (Fig. 2). Use of a single site reduces logistical challenges, but the results may not be generalisable to other environments with a different location, business models and case-mix. For example, the record review conducted in the Department of Veterans Affairs, which caters to a population that is predominantly male, may not be generalised to clinic settings and patient populations outside the Veterans Affairs system [50].

Appropriateness of eye care was generally measured as compliance against scientific evidence or consensus with clinical experts in the field with around two-thirds of the articles having measured eye care appropriateness against recommendations from clinical practice guidelines (*n* = 38) and 16% having used experts’ opinions (*n* = 9).

A small number of studies measured eye care appropriateness against expert care rather than against clinical practice guidelines, where the same patients are examined twice, once by the practitioners and once by experts [36, 135, 143].

Eye care appropriateness results are summarized in Table 2. It is important to note at the outset that the timing (e.g. once during a period, at the diagnosis visit, etc.), type of visits (e.g. first visit, follow-up visit, etc.), the health professions and settings assessed, and the method used to collect the data (e.g. record review) vary between studies (see Table 2) and may confound the appropriateness of eye care results.

**Table 2:** Appropriateness of eye care by domain of care. Numbers are percentage of encounters with appropriate care (number of quality indicators). If more than one quality indicator was assessed, the percentage of encounters with appropriate care is presented as a range of percentage. NZ = New Zealand, A&E = accident and emergency, N/A = not applicable as no specific timing was measured

Country	Year	Health Practitioner	Timing	Domain of care						Author (reference)
				History taking	Physical examination	Management	Recall period	Referral	Patient education	
Glaucoma										
UK	2013	Ophthalmologist	All visits (at least up to 17.5 years)		0,87% (1)^a^					Fung et al. [26]
UK	2012	Optometrist	First visit		74–100% (6)	96% (1)				Chawla et al. [27]
			First follow-up visit	88% (1)	94–100% (3)		92% (2)			
		Ophthalmologist	First visit		10–100% (6)	100% (1)				
			First follow-up visit	24% (1)	8–100% (3)		66–86% (2)			
UK	2012	Optometrist	Referral letter for glaucoma diagnosis					70% (1)^b^ 4–99% (6)^c^		Khan et al. [29]
UK	2012	Optometrist	Results of interview	77% (1)	19–98% (4)					Theodossiades et al. [31]
			First visit of standardised patient	41% (1)	3–100% (4)					
UK	2011	Ophthalmologist	N/A			23% (1)				Stead et al. [32]
UK	2009	Optometrist	Referral letter for glaucoma diagnosis					27–100% (14)^c^		Scully et al. [35]
UK	2012	Optometrist	First full visit		91–98% (1)	97% (1)		87% (1)2		Marks et al. [36]
UK	2011	Optometrist	All follow-up visits		96% (1)	99% (1)	93% (1)			Ho and Vernon [37]
UK	2011	Optometrist	Referral letter for glaucoma diagnosis					25% (1)^b^		Shah and Murdoch [38]
UK	2010	Optometrist	All visits			93% (1)	86% (1)			Syam et al. [39]
UK	2010	Optometrist	Referral letter for glaucoma diagnosis					37% (1)^b^ 72–99% (3)^c^		Lockwood et al. [40]
UK	2007	Optometrist	First visit			85% (1)				Azuara-Blanco et al. [41]
		Ophthalmologist	First visit			83% (1)				
UK	2006	Optometrist	Referral letter for glaucoma diagnosis					45% (1)^b^		Patel et al. [42]
UK	2006	Optometrist	All follow-up visit		62–98% (5)	72–97% (5)	79% (1)			Banes et al. [43]
		Associate specialists	All follow-up visit		54–100% (5)	71–99% (5)	73% (1)			
USA	2016	Ophthalmologist	All follow-up visits			68% (1)				Solano-Moncada et al. [45]
USA	2016	Ophthalmologist & optometrist	All visits within 2 years after glaucoma diagnosis		27–74% (2)					Elam et al. [46]
USA	2015	Resident ophthalmologist	Third (or more) follow-up visit	88% (1)	62–100% (5)	74% (1)				Zebardast et al. [48]
		Faculty ophthalmologist	Third (or more) follow-up visit	100% (1)	87–100% (5)	100% (1)				
USA	2013	Resident ophthalmologist	First follow-up visit	49–97% (5)	93–100% (4)	82–100% (6)	96–97% (2)	16% (1)	5% (1)	Ong et al. [50]
USA	2012	Ophthalmologist & optometrist	All visits within 3 years after glaucoma or glaucoma suspect diagnosis		12–34% (2)					Swamy et al. [51]
USA	2007	Ophthalmologist	First claim for a prostaglandin prescription		50–90% (5)	19% (1)	100% (1)		38% (1)	Quigley et al. [52]
USA	2006	Ophthalmologist	All visits within 5 years before surgery for glaucoma		49% (1)					Coleman et al. [54]
Australia & NZ	2015	Optometrist (Australia)	N/A	99% (1)	25–100% (10)					Zangerl et al. [56]
		Optometrist (NZ)	N/A	100% (1)	27–100% (10)					
Australia & NZ	2008	Ophthalmologist	N/A		13–96% (4)					Liu [59]
Scotland	2015	Optometrist	Referral letter for glaucoma diagnosis BEFORE guidelines published					62% (1)^b^ 33–85% (3)^c^		El-Assal et al. [61]
			Referral letter for glaucoma diagnosis AFTER guidelines published					76% (1)^b^ 76–81% (3)^c^		
Scotland	2009	Optometrist	Referral letter for glaucoma progression BEFORE guidelines published					18% (1)^b^ 2–94% (7)^c^		Ang et al. [62]
			Referral letter for glaucoma progression AFTER guidelines published					32% (1)^b^ 24–93% (7)^c^		
Canada	2014	Ophthalmologist	Referral letter for glaucoma diagnosis					10–100% (16)^c^		Cheng et al. [64]
		Optometrist	Referral letter for glaucoma diagnosis					7–100% (16)^c^		
Germany	2008	Ophthalmologist	N/A			96% (1)				Vorwerk et al. [65]
Singapore	2008	Ophthalmologist	N/A		75–93% (2)					Ang et al. [67]
Diabetic retinopathy										
Australia	2011	Optometrist	N/A					83–99% (2)^b^		Slater and Chakman [69]
Australia	2011	Optometrist	N/A	43–96% (6)	23–89% (2)		6–98% (12)^d^			Ting et al. [71]
Australia	2010	Ophthalmologist	N/A	41–55% (4)	49–90% (2)	56–94% (2)	38–71% (10)^d^			Yuen et al. [72]
NZ	2012	Optometrist	Fundus screening visit					60% (1)^b^		Hutchins et al. [74]
USA	2012	Ophthalmologist & optometrist	N/A		71% (1)					Chou et al. [76]
USA	2010	Resident ophthalmologist	First ever diabetic retinopathy examination	41–57% (5)	0–100% (7)	70–79% (2)	69–70% (2)		0–27% (3)	Tseng et al. [78]
Hong Kong	2016	General practitioner	N/A		33% (1)		27% (1)			Wong et al. [80]
Bahrain	2014	General practitioner at general practitioner clinic	All follow-up visits within previous 12 months		0% (1)^e^					Al-Ubaidi et al. [82]
		General practitioner at diabetes care clinic	All follow-up visits within previous 12 months		87% (1)^e^					
Switzerland	2013	General practitioner	First hospitalisation		31% (1)^e^					Burgmann et al. [84]
UK	2011	General practitioner	Second diabetic visit		71% (1)^e^					Mc Hugh et al. [86]
Brazil	2007	General practitioner	N/A		34–87% (2)^e^					Preti et al. [88]
Age-related Macular Degeneration										
Italy	2016	Ophthalmologist	N/A			44% (1)				Parodi et al. [93]
Turkey	2015	Ophthalmologist	N/A			23% (1)				Muhammed et al. [95]
UK	2013	Ophthalmologist & optometrist	N/A	21–32% (2)		28–70% (5)			49% (1)	Lawrenson and Evans [100]
USA	2008	Ophthalmologist	N/A			76% (1)				Charkoudian et al. [103]
Cataract										
UK	2011	Ophthalmologist	N/A			51–99% (3)				Gomaa and Liu [105]
UK	2009	Optometrist	Referral letter for cataract surgery					0–100% (10)^c^		Park et al. [107]
		General practitioner	Referral letter for cataract surgery					0–100% (10)^c^		
UK	2006	Optometrist	Referral letter for cataract surgery					48% (1)^c^		Lash et al. [109]
USA	2009	Resident ophthalmologist	Preoperative care visits for first cataract surgery	73–100% (4)	59–100% (9)	0–100% (9)				Niemiec et al. [111]
			All postoperative follow-up visits for first cataract surgery	14–78% (6)	77–100% (7)	98% (1)	98% (1)	43% (1)^b^	98% (1)	
Preventative eye care										
UK	2009	Optometrist	First visit	95% (1)	0–100% (5)					Shah et al. [115]
UK	2009	Optometrist	First visit	26–87% (8)	24–99% (10)	29% (1)				Shah et al. [118]
UK	2008	Optometrist	First visit	1–100% (14)	59–100% (8)	14–80% (6)				Shah et al. [120]
Australia	2015	Optometrist	N/A	47–55% (2)		62–80% (2)				Downie and Keller [129]
Dry eye										
Australia	2013	Optometrist	N/A		4–93% (3)					Downie et al. [132]
USA	2010	Ophthalmologist	Initial diagnosis visit BEFORE guidelines revised	6–99% (12)	6–100% (12)	5–90% (5)		48% (1)^b^	47–89% (3)	Lin et al. [134]
			Initial diagnosis visit AFTER guidelines revised	6–100% (16)	6–100% (13)	0–100% (7)		33% (1)^b^	33–89% (4)	
All ocular conditions at A&E										
UK	2007	Optometrist	First visit					91% (1)		Hau et al. [135]
Amblyopia										
USA	2013	Ophthalmologist	Initial visit			12–24% (2)				Jin et al. [138]
Esotropia										
USA	2010	Ophthalmologist	Initial esotropia evaluation	64% (4)^f^	99.6% (6)^f^	94% (4)^f^			94% (2)^f^	Gupta et al. [140]
				70% (4)^g^	90% (6)^g^	94% (4)^g^			94% (4)^g^	
Non-infectious uveitis										
USA	2011	Ophthalmologist & rheumatologist	All visits since initial diagnosis			12–23% (2)				Nguyen et al. [142]

^a^Fung et al. [26] reported 0 and 87% compliance for frequency of visual fields examination against two sets of glaucoma guidelines, the European Glaucoma Society (EGS) [24] and the United Kingdom’s National Institute for Health and Clinical Excellence (NICE) guidelines [25], respectively. ^b^Percentage of appropriateness of referral to relevant health practitioners. ^c^Percentage of appropriate content of the referral letters. ^d^‘’Recall period’ and ‘referral’ were assessed by the same set of case vignettes [71, 72]. ^e^Percentage of diabetic patients who visited general practitioners and were arranged a diabetic retinopathy screening by ophthalmologists. ^f^Mean appropriate care measured against guidelines published by American Academy of Ophthalmology (AAO) in 2002. Appropriate care was defined as documentation of 50% or more of the specific parameters listed for each quality indicator. ^g^Mean appropriate care measured against guidelines published by NICE in 2007. Appropriate care was defined as documentation of 50% or more of the specific parameters listed for each quality indicator

**Fig. 3:**
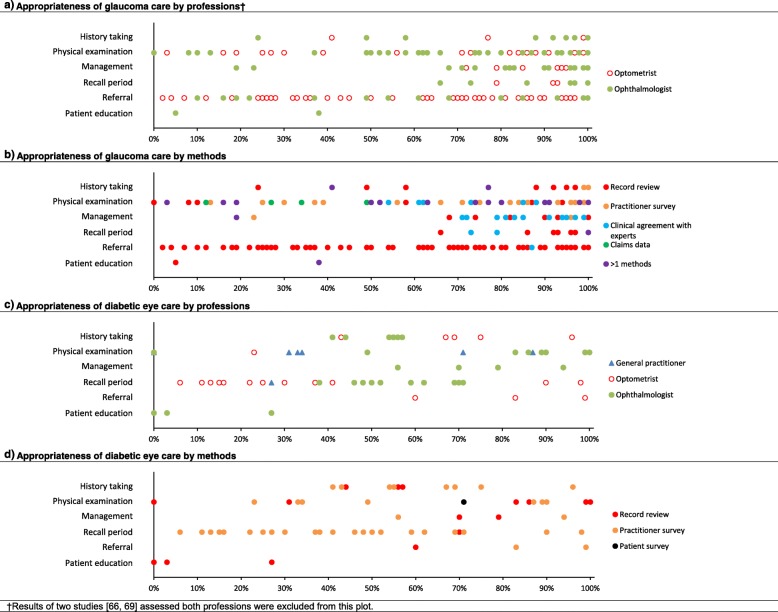
Appropriateness of eye care for glaucoma (**a**, **b**) and diabetic retinopathy (**c**, **d**) for various domains of care by profession (**a**, **c**) and methods (**b**, **d**). All quality indicators from the included studies were pooled together. Each data point represented the percentage of compliance against a quality indicator. **a** Overall, the appropriateness of glaucoma care ranged widely from 2 to 100%. The appropriateness of glaucoma delivered by optometrists and ophthalmologists appeared similar. **b** When appropriateness of glaucoma care was assessed using clinical agreement with experts, care was delivered appropriately at least 50% of the time. The appropriateness of glaucoma care assessed using other methods ranged more widely. **c**, **d** The appropriateness of diabetic eye care ranged widely from 0 to 100%. The wide range and the relatively small number of studies measuring appropriateness of diabetic eye care limited our ability to detect obvious patterns in individual domains for diabetes care

Twenty-eight studies reporting on eye care appropriateness in glaucoma screening, glaucoma suspects and/or glaucoma patients were included. In more than half of the studies (15 of 28), the appropriateness of glaucoma care was measured via a review of hospital records. Appropriate ‘management’ and ‘recall period’ for glaucoma were reported most of the time, whereas ‘physical examination’ and ‘referral’ for glaucoma were not delivered as appropriately at times (Fig. 3a and b). Overall, the appropriateness of glaucoma care ranged widely from 2 to 100%. The appropriateness of glaucoma care assessed using clinical agreement with experts was the only method where appropriate care was delivered consistently at least 50% of the time. Although studies investigated the appropriateness of glaucoma delivered by optometrists and ophthalmologists, no obvious differences between professions were noted.

Eleven studies have reported on appropriateness of eye care delivery in diabetic patients. Overall, diabetes eye care compliance also ranged widely from 0 to 100%. That wide range and the relatively small number of studies available makes it challenging to detect obvious patterns in individual domains for diabetes care (Fig. 3c and d). For example, only a single study with three quality indicators sampled the appropriateness of ‘patient education’ in diabetes eye care at a single site and reported a below 50% appropriateness of ‘patient education’ overall.

Appropriateness of eye care delivery has been measured for cataract, age-related macular degeneration, preventative eye care and five other ocular conditions in 18 separate articles (Table 2). Eye care appropriateness also ranged widely in those studies, for example from 0 to 100% for dry eye care [134] and for the referral of cataract surgery [107].

Very few studies examined or reported on factors that can modulate appropriateness of eye care delivery. Modifiable factors that have been shown to impact appropriateness of eye care delivery include data entry system (i.e. electronic or paper records) [134], health insurance coverage [76], higher eye care provider density [76], awareness of clinical practice guidelines availability [142], procedural confidence and therapeutic endorsement of optometrists [56] and specialty training conducted in a supportive environment [43]. Non-modifiable factors that may impact appropriateness of eye care include the severity of patients’ eye condition [71], patient’s age and ethnicity [54], and practitioner’s age [72, 129], gender [129] and years of experience [88]. These factors must therefore be measured and controlled for in any future studies assessing the appropriateness of eye care delivery.

## Discussion

This systematic literature review summarises studies reporting the process of eye care delivery in many different countries using existing published evidence and/or experts’ practice to measure appropriateness of eye care. The appropriateness of eye care delivered was found to vary widely for the most commonly reported conditions (glaucoma and diabetic eye care) from 0 to 100%. Appropriate ‘management’ and ‘recall period’ for glaucoma were observed. Record review was most commonly used to assess the appropriateness of eye care delivery; this may be explained by the ease of administration and low cost associated with this method, especially when conducted at a single site.

The methodological quality was rated as moderate on average across all methods. Different quality assessment tools were used for to appraise studies with different study design, where some criteria were the same between tools. With consideration of the variety of the study designs and the total numbers of included studies, it was considered beneficial to use a modified quality assessment tool with all questions sourced from existing validated critical appraisal tools (Additional file 2). The quality of the included studies should not be different when different tools are used, when the studies are assessed against the same questions from the existing validated critical appraisal tools.

Comparison of the overall appropriateness of eye care versus the appropriateness for individual domains of eye care between studies presented some challenges for the following reasons:

1. *Differences in the number of quality indicators used.* Seven quality indicators were used in the Zebardast et al. [48] study, but 19 quality indicators were used by Ong et al. [50] Although both studies assessed appropriateness of eye care against the same glaucoma guidelines, the overall result cannot be easily compared, unless this is done by comparing appropriateness of care of individual quality indicators used by both studies.
2. *Differences in eligibility criteria and time frame of quality indicators.* Quigley et al. [52] assessed whether practitioners have performed gonioscopy at least once within the previous 6 years for all patients with open-angle glaucoma and found that appropriate care was delivery only 50% of the time. Conversely, Ong et al. [50] reported 90% appropriate care for performing gonioscopy on indication. A possible conclusion may be that practitioners in the latter study performed much better than in the former. However, careful observation of the study population characteristics reveals that this appropriateness of care results simply reflects how often practitioners perform gonioscopy in open angle glaucoma in the first instance and use of gonioscopy in cases with a suspicious angle in the latter study.
3. *Differences in time interval*. Chawla et al. [27] assessed both planned and actual review interval for glaucoma against the guidelines whereas Ong et al. [50] only assessed if the planned follow-up complied with guidelines.
4. *Different aspects of the quality indicator are assessed.* Appropriateness of ‘referral’ can be considered in terms of the appropriateness of the referral criteria, the timing of the referral or in terms of the appropriateness and contents of referral letters. Appropriateness of referral often describes whether patients were referred to the correct people or facilities. Appropriateness and contents of referral letters typically considers if the referral letters contained the required information, according to guidelines or specialist’s opinions. However, the percentage of appropriate care of these two aspects may not directly be comparable. Appropriateness of referral pathway or criteria is not necessarily equivalent to an appropriate referral letter and vice versa. For example, Ang et al. [62] reported that the appropriateness of referral letters from optometrists referring for glaucoma progression was 32% whereas the appropriateness and contents of their referral letters exhibited 24–93% compliance against the seven quality indicators used.
5. *Differences in quality indicator weighing.* Most studies weighed all quality indicators evenly, but some assigned different weightings for different quality indicators. Quigley et al. [52] assigned weighting (0, 1, 2 or 3) according to the imputed importance of individual items. Gupta et al. [140] defined appropriate care as the practitioners documenting 50% or more of the sub-indicators listed for each element. For example, once 2 or more of the 4 sub-indicators (frequency of deviation, date of onset, and presence of diplopia or squint) of ocular signs and symptoms were documented, this quality indicator was counted as compliant.

The findings of this systematic review are limited by the lack of a standardised method to measure and report the appropriateness of eye care delivery. The extent to which eye care appropriateness may have been under or overestimated may be significantly influenced by the choice of method used to assess care delivery in these studies. Two-thirds of the included articles measured compliance against recommendations from clinical practice guidelines, which are likely to have been developed using similar evidence sources. In this review, this is likely to have manifested as reporting the appropriateness of eye care according to a somewhat narrow evidence base. However, clinical practice guidelines are primarily developed for and made available to clinicians for the purposes of guiding evidence-based care, which lends credibility to their use as a compliance tool. In addition, studies conducted in one country might not reflect the appropriateness of eye care received in a different country where the health care and education systems, values and expectations could be significantly different [144]. Given that and the diversity of countries where eye care appropriateness has been measured, the generalisability of the various reported findings to other countries is uncertain.

## Conclusion

Studies reporting the appropriateness of eye care delivery in Australia and other developed mainly English-speaking countries, indicated a wide range of appropriateness of care delivery, for glaucoma and diabetic eye care. Existing eye-related studies have assessed a single condition, a sample from a single centre or a single domain of care even as specific as only one examination technique such as gonioscopy. Consequently, none of the studies identified in the literature review attempted to measure the overall appropriateness of care provided in eye care. One important purpose of measuring appropriateness of care is to help policy makers to allocate limited health resources. Future research would benefit from a more comprehensive approach where appropriateness of eye care delivery is measured across multiple conditions with a single methodology to guide priorities within eye care delivery and monitor quality improvement initiatives.

## Additional files

Additional file 1: Full electronic search strategy for Medline. (DOCX 15 kb)

Additional file 2: Quality assessment tool. (DOCX 22 kb)

Additional file 3: Results of quality appraisal of included studies. (DOCX 52 kb)

## Data Availability

The datasets generated and/or analysed during the current study are available in the Zenodo repository, DOI: (10.5281/zenodo.2597710).

## Abbreviation

A&E: Accident and emergency
AAO: American Academy of Ophthalmology
CASP: Critical Appraisal Skills Programme
CINAHL: Cumulative Index to Nursing and Allied Health Literature
EGS: European Glaucoma Society
EPHPP: Effective Public Health Practice Project
JBI: Joanna Briggs Institute
N/A: Not applicable
NICE: National Institute for Health and Clinical Excellence
NIH: National Institutes of Health
NZ: New Zealand
PRISMA: Preferred Reporting Items for Systematic reviews and Meta-Analyses
UK: United Kingdom
USA: United States of America
