# Supplementary material for: Systematic review of the appropriateness of eye care delivery in eye care practice
Source: BMC Health Serv Res. 2019 Sep 6;19:646. doi: 10.1186/s12913-019-4493-3 (PMC6731572; doi:10.1186/s12913-019-4493-3)
Supplement: Supplementary file 1 — Full electronic search strategy for Medline. (DOCX 15 kb) [file 12913_2019_4493_MOESM1_ESM.docx]

| ***Additional File 1. Full electronic search strategy for Medline*** |
| --- |
| Limit applied “English” “2006-present” |
| 1. “Optometr*” 2. “Ophthalmolog*” 3. “General practitioner*” 4. “Orthopt*” 5. “Ophthalmic nurse*” 6. “Ophthalmic practitioner*” 7. 1 OR 2 OR 3 OR 4 OR 5 OR 6 8. Exp”Quality of Health Care” 9. Exp Glaucoma 10. Exp diabetic retinopathy 11. Exp refractive errors 12. Exp macular degeneration 13. Exp cataract 14. 9 OR 10 OR 11 OR 12 OR 13 15. 7 AND 8 AND 14 |
