# Supplementary material for: Systematic review of the appropriateness of eye care delivery in eye care practice
Source: BMC Health Serv Res. 2019 Sep 6;19:646. doi: 10.1186/s12913-019-4493-3 (PMC6731572; doi:10.1186/s12913-019-4493-3)
Supplement: Supplementary file 2 — Quality assessment tool. (DOCX 22 kb) [file 12913_2019_4493_MOESM2_ESM.docx]

| Additional File 2. Quality assessment tool***.*** |  |
| --- | --- |
| Answer **Yes, No, NA** or **Unclear** for each of the following questions. Please specify the reason(s) for ‘**No**’, ‘**NA**’ and ‘**Unclear**’ or whenever necessary. | |
| - *Reporting (possibility to repeat the study)*   Reporting | **Question source**s |
| Q1)      Is there a clear statement of the aims of the research? | Dyba *et al.,*^1^ CASP^2^ and NIH^3^ |
| Q2)      Is there an adequate description of the context in which the research was carried out? (e.g. current understanding, background information about the topic) | Dyba *et al.*^1^ |
| Q3)      Is the eligibility (e.g. disease status) of the tested population clearly described? | NIH^3^ |
| Q4)      Were the methods for measurement described in sufficient detail? | CASP^2^ |
| Q5)      Is there a clear statement of findings? | CASP^2^ |
| - *Validity of data collection tools and the analysis methods, and hence trustworthiness of the findings* |  |
| Selection bias |  |
| Q6)      Are the individuals selected to participate in the study likely to be representative of the target population? | JBI^4^ and NIH^3^ |
| Q7)      Was the participation rate of eligible persons at least 50%? (If applicable) | NIH^3^ |
| Q8)      Was the sample size justification provided or was the sample size adequate? (i.e. large sample size may not necessarily need to calculate sample size) | NIH^3^ |
| Study design |  |
| Q9)      Was the study described as randomised and the method of randomisation described and appropriate? | EPHPP^5^ |
| Q10)   Was there a control group with which to compare interventions? (If applicable) (e.g. historical control) | Dyba *et al.*^1^ and EPHPP^5^ |
| Blinding |  |
| Q11)   Was (were) the outcome assessor(s) blinded to the intervention or exposure status of participants? | CASP^2^, NIH^3^ and EPHPP^5^ |
| Q12)   Were the study participants blinded to the research question? | EPHPP^5^ |
| Q13)   Was there an independent, blind comparison between the subject of interest and an appropriate reference standard? (if applicable) | CASP^2^ |
| Data collection tools |  |
| Q14)   Was the care quality measured reliably? (e.g. use tested data/piloted data collection tool, i.e. tested survey, trained surveyors) | JBI^4^ and NIH^3^ |
| Q15)   Were objective, standard criteria used for the measurement of care quality? (e.g. based on guidelines, experts, consultant ophthalmologist) | CASP ^2^ |
| Analysis |  |
| Q16)   Was the data analysis sufficiently rigorous? (e.g. p-value? statistical significance calculated?) (Dyba *et al.* ^1^, CASP ^2^) | Dyba *et al.,*^1^ JBI^4^ and EPHPP^5^ |
| Confounders |  |
| Q17)     Were key potential confounding variables identified and accounted for? (e.g. mentioned in limitations, measured and adjusted statistically for their impact, i.e. multi-variate analysis) (If applicable) (NIH ^3^) | NIH^3^, JBI^4^ and EPHPP^5^ |
| ^1^CASP=Critical Appraisal Skills Programme; NIH=National Institutes of Health; JBI=Joanna Briggs Institute; EPHPP=Effective Public Health Practice Project. | |

**REFERENCES**

1. Dyba TD, T.; Hanssen, G. K. . Applying Systematic Reviews to Diverse Study Types: An Experience Report. First International Symposium on Empirical Software Engineering and Measurement (ESEM 2007); Madrid: IEEE Xplore; 2007. p. 225-234.

2. Critical Appraisal Skills Programme. CASP diagnostic checklist. 2013. <http://www.casp-uk.net/checklists> (accessed 03rd Mar 2017).

3. National Institutes of Health. Quality Assessment Tool For Observational Cohort And Cross-Sectional Studies. 2014.

4. Joanna Briggs Institute. JBI Critical Appraisal Checklist For Studies Reporting Prevalence Data. 2014.

5. Effective Public Health Practice Project. Quality assessment tool for quantitative studies. 2010. <http://www.ephpp.ca/tools.html> (accessed 03rd Mar 2017).
