# Supplementary material for: Systematic review of the appropriateness of eye care delivery in eye care practice
Source: BMC Health Serv Res. 2019 Sep 6;19:646. doi: 10.1186/s12913-019-4493-3 (PMC6731572; doi:10.1186/s12913-019-4493-3)
Supplement: Supplementary file 3 — Results of quality appraisal of included studies. (DOCX 52 kb) [file 12913_2019_4493_MOESM3_ESM.docx]

| **Additional File 3. Results of quality appraisal of included studies.** A&E=accident and emergency | | | | | | | | | | | | | | | | | | |
| --- | --- | --- | --- | --- | --- | --- | --- | --- | --- | --- | --- | --- | --- | --- | --- | --- | --- | --- |
| **Author** | **Reporting** | | | | | **Selection bias** | | | **Study design** | | **Blinding** | | | **Data collection tools** | | **Analysis** | **Confounders** | **Overall quality‡** |
|  | Q1† | Q2† | Q3† | Q4† | Q5† | Q6† | Q7† | Q8† | Q9† | Q10† | Q11† | Q12† | Q13† | Q14† | Q15† | Q16† | Q17† |  |
| **Glaucoma** |  |  |  |  |  |  |  |  |  |  |  |  |  |  |  |  |  |  |
| Ang *et al.*^1^ | Yes | Yes | Yes | Yes | Yes | Yes | NA | No | No | Yes | No | Yes | NA | No | Yes | Yes | No | Moderate |
| Ang *et al.*^2^ | Yes | Yes | Yes | Yes | Yes | Yes | Yes | No | NA | NA | NA | NA | NA | No | Yes | Yes | NA | Strong |
| Azuara-Blanco *et al.*^3^ | Yes | Yes | Yes | Yes | Yes | Yes | Yes | Yes | Yes | Yes | NA | No | Yes | No | Yes | Yes | Yes | Strong |
| Banes *et al.*^4^ | Yes | Yes | No | Yes | Yes | No | NA | Yes | No | Yes | No | No | Yes | No | Yes | Yes | Yes | Moderate |
| Chawla *et al.*^5^ | Yes | Yes | Yes | No | Yes | No | NA | No | No | Yes | NA | NA | NA | Yes | Yes | No | No | Weak |
| Cheng *et al.*^6^ | Yes | No | Yes | Yes | Yes | Yes | NA | No | NA | NA | No | Yes | NA | No | Yes | Yes | Yes | Moderate |
| Coleman *et al.*^7^ | Yes | Yes | Yes | Yes | Yes | Yes | NA | Yes | NA | NA | NA | NA | NA | NA | Yes | Yes | Yes | Strong |
| Elam *et al.*^8^ | Yes | No | Yes | Yes | Yes | Yes | NA | Yes | NA | NA | NA | NA | NA | NA | Yes | Yes | Yes | Strong |
| El-Assal *et al.*^9^ | Yes | Yes | Yes | Yes | Yes | Yes | NA | Yes | No | Yes | No | Yes | NA | Yes | Yes | Yes | Yes | Strong |
| Fung *et al.*^10^ | Yes | Yes | Yes | Yes | Yes | No | NA | No | NA | NA | NA | NA | NA | Yes | Yes | Yes | Yes | Strong |
| Ho and Vernon ^11^ | Yes | Yes | Yes | Yes | Yes | No | NA | No | NA | NA | No | Yes | No | No | Yes | Yes | Yes | Moderate |
| Khan *et al.*^12^ | Yes | Yes | Yes | No | Yes | No | NA | No | NA | NA | NA | NA | NA | No | Yes | Yes | Yes | Moderate |
| Liu ^13^ | Yes | Yes | Yes | Yes | Yes | Yes | Yes | Yes | NA | NA | NA | NA | NA | Yes | No | Yes | Yes | Strong |
| Lockwood *et al.*^14^ | Yes | Yes | Yes | No | Yes | No | NA | No | NA | NA | NA | NA | No | No | Yes | Yes | Yes | Weak |
| Marks *et al.*^15^ | Yes | Yes | Yes | Yes | Yes | Yes | NA | No | NA | NA | No | No | Yes | No | Yes | Yes | Yes | Moderate |
| Ong *et al.*^16^ | Yes | Yes | Yes | Yes | Yes | Yes | NA | No | NA | NA | NA | NA | NA | Yes | Yes | Yes | Yes | Strong |
| Patel *et al.*^17^ | Yes | Yes | Yes | No | Yes | Yes | NA | No | No | No | No | No | No | NA | Yes | No | No | Weak |
| Quigley *et al.*^18^ | Yes | Yes | Yes | Yes | Yes | Yes | NA | No | No | NA | NA | NA | NA | Yes | Yes | Yes | Yes | Moderate |
| Scully *et al.*^19^ | Yes | Yes | Yes | Yes | Yes | Yes | NA | No | NA | NA | NA | NA | NA | No | Yes | Yes | No | Moderate |
| Shah and Murdoch^20^ | Yes | Yes | Yes | Yes | Yes | No | NA | No | NA | Yes | NA | Yes | No | NA | Yes | No | Yes | Moderate |
| Solano-Moncada *et al.*^21^ | Yes | Yes | Yes | Yes | Yes | No | NA | No | NA | NA | NA | NA | NA | No | Yes | Yes | Yes | Moderate |
| Stead *et al.*^22^ | Yes | Yes | No | No | Yes | Yes | Yes | Yes | NA | NA | NA | NA | NA | No | Yes | Yes | Yes | Moderate |
| Swamy *et al.*^23^ | Yes | Yes | Yes | Yes | Yes | Yes | NA | Yes | NA | NA | NA | NA | NA | NA | Yes | Yes | Yes | Moderate |
| Syam *et al.*^24^ | Yes | Yes | Yes | Yes | Yes | Yes | NA | Yes | NA | NA | No | No | NA | Yes | Yes | Yes | Yes | Strong |
| Theodossiades *et al.*^25^ | Yes | Yes | Yes | Yes | Yes | No | No | No | No | Yes | No | Yes | NA | Yes | Yes | Yes | Yes | Moderate |
| Vorwerk *et al.*^26^ | Yes | Yes | No | Yes | Yes | No | No | No | NA | NA | NA | NA | NA | Yes | Yes | Yes | Yes | Moderate |
| Zangerl *et al.*^27^ | Yes | Yes | Yes | Yes | Yes | No | No | Yes | NA | NA | NA | NA | NA | No | Yes | Yes | Yes | Moderate |
| Zebardast *et al.*^28^ | Yes | Yes | Yes | Yes | Yes | No | NA | No | NA | Yes | No | No | NA | Yes | Yes | Yes | Yes | Moderate |
| **Diabetic retinopathy** | |  |  |  |  |  |  |  |  |  |  |  |  |  |  |  |  |  |
| Al-Ubaidi *et al.*^29^ | Yes | Yes | Yes | Yes | Yes | Yes | NA | No | NA | NA | NA | NA | NA | Yes | Yes | Yes | Yes | Strong |
| Burgmann *et al.*^30^ | Yes | Yes | Yes | No | Yes | Yes | NA | No | NA | NA | NA | NA | NA | No | Yes | Yes | Yes | Moderate |
| Chou *et al.*^31^ | Yes | Yes | Yes | Yes | Yes | No | NA | Yes | NA | NA | Yes | Yes | NA | Yes | Yes | Yes | Yes | Strong |
| Hutchins *et al.*^32^ | Yes | Yes | Yes | Yes | Yes | Yes | NA | No | NA | NA | NA | NA | NA | No | Yes | Yes | Yes | Strong |
| Mc Hugh *et al.*^33^ | Yes | Yes | Yes | Yes | Yes | Yes | Yes | Yes | NA | Yes | NA | NA | NA | yes | Yes | Yes | Yes | Strong |
| Preti *et al.*^34^ | Yes | Yes | No | No | Yes | No | No | No | NA | NA | NA | NA | NA | No | Yes | Yes | No | Weak |
| Slater and Chakman ^35^ | Yes | Yes | No | No | Yes | No | No | Yes | NA | NA | NA | NA | NA | No | Yes | No | No | Weak |
| Ting *et al.*^36^ | Yes | Yes | Yes | Yes | Yes | Yes | Yes | Yes | NA | NA | NA | NA | NA | Yes | Yes | Yes | Yes | Strong |
| Tseng *et al.*^37^ | Yes | Yes | Yes | Yes | Yes | Yes | NA | No | NA | NA | NA | NA | NA | No | Yes | Yes | Yes | Strong |
| Wong *et al.*^38^ | Yes | Yes | Yes | Yes | Yes | No | No | Yes | NA | NA | NA | NA | NA | Yes | Yes | Yes | Yes | Strong |
| Yuen *et al.*^39^ | Yes | Yes | Yes | Yes | Yes | No | Yes | Yes | NA | NA | NA | NA | NA | Yes | Yes | Yes | Yes | Strong |
| **Age-related macular degeneration** | | | |  |  |  |  |  |  |  |  |  |  |  |  |  |  |  |
| Charkoudian *et al.*^40^ | Yes | Yes | Yes | Yes | Yes | No | Yes | No | NA | NA | NA | NA | NA | No | Yes | Yes | Yes | Moderate |
| Lawrenson and Evans ^41^ | Yes | Yes | Yes | Yes | Yes | No | No | No | NA | NA | NA | NA | NA | Yes | No | Yes | No | Weak |
| Muhammed *et al.*^42^ | Yes | Yes | Yes | Yes | Yes | No | No | No | NA | NA | NA | NA | NA | No | Yes | Yes | Yes | Moderate |
| Parodi *et al.*^43^ | Yes | Yes | Yes | Yes | Yes | Yes | Yes | No | NA | NA | Yes | No | NA | No | Yes | Yes | No | Moderate |
| **Cataract** |  |  |  |  |  |  |  |  |  |  |  |  |  |  |  |  |  |  |
| Gomaa and Liu ^44^ | Yes | Yes | Yes | No | Yes | No | Yes | No | NA | NA | NA | NA | NA | No | Yes | Yes | No | Weak |
| Lash *et al.*^45^ | Yes | Yes | Yes | Yes | Yes | Yes | NA | No | NA | NA | NA | No | No | Yes | Yes | Yes | No | Moderate |
| Park *et al.*^46^ | Yes | Yes | Yes | Yes | Yes | Yes | NA | Yes | No | Yes | No | Yes | NA | No | Yes | Yes | Yes | Strong |
| Niemiec *et al.*^47^ | Yes | Yes | Yes | Yes | Yes | Yes | NA | No | NA | NA | NA | NA | NA | No | Yes | Yes | Yes | Strong |
| **Preventative eye care** | | |  |  |  |  |  |  |  |  |  |  |  |  |  |  |  |  |
| Downie and Keller ^48^ | Yes | Yes | Yes | Yes | Yes | No | No | No | NA | NA | NA | NA | NA | Yes | No | Yes | Yes | Moderate |
| Shah *et al.*^49^ | Yes | Yes | Yes | Yes | Yes | No | No | No | NA | NA | NA | No | NA | Yes | Yes | Yes | Yes | Moderate |
| Shah *et al.*^50^ | Yes | Yes | Yes | Yes | Yes | No | No | No | NA | NA | NA | No | NA | Yes | Yes | Yes | Yes | Moderate |
| Shah *et al.*^51^ | Yes | Yes | Yes | Yes | Yes | No | No | No | NA | NA | NA | No | NA | Yes | Yes | Yes | Yes | Moderate |
| **Dry eye** |  |  |  |  |  |  |  |  |  |  |  |  |  |  |  |  |  |  |
| Lin *et al.*^52^ | Yes | No | Yes | No | Yes | No | NA | No | NA | NA | NA | NA | NA | No | Yes | Yes | Yes | Weak |
| Downie *et al.*^53^ | Yes | Yes | Yes | Yes | Yes | No | No | No | NA | NA | NA | NA | NA | Yes | Yes | Yes | No | Moderate |
| **All ocular conditions at A&E** | | |  |  |  |  |  |  |  |  |  |  |  |  |  |  |  |  |
| Hau *et al.*^54^ | Yes | No | Yes | Yes | Yes | No | NA | Yes | NA | NA | NA | No | Yes | NA | Yes | Yes | No | Moderate |
| **Amblyopia** |  |  |  |  |  |  |  |  |  |  |  |  |  |  |  |  |  |  |
| Jin *et al.*^55^ | Yes | Yes | Yes | Yes | Yes | Yes | NA | No | NA | Yes | NA | NA | NA | No | Yes | Yes | No | Moderate |
| **Esotropia** |  |  |  |  |  |  |  |  |  |  |  |  |  |  |  |  |  |  |
| Gupta *et al.*^56^ | No | No | Yes | No | Yes | Yes | NA | No | NA | NA | NA | NA | NA | No | Yes | Yes | Yes | Weak |
| **Non-infectious uveitis** | |  |  |  |  |  |  |  |  |  |  |  |  |  |  |  |  |  |
| Nguyen *et al.*^57^ | Yes | Yes | Yes | Yes | Yes | Yes | NA | No | NA | NA | NA | NA | NA | No | Yes | No | Yes | Moderate |
| †Questions referred to questions listed in the quality assessment tool listed in **Additional file 1.**  ‡If less than 60% criteria in the quality assessment tool were met, quality was scored as weak; it was scored moderate if 60-79% were met and strong if 80%-100% were met. | | | | | | | | | | | | | | | | | | |

**REFERENCES**

1. Ang GS, Ng WS & Azuara-Blanco A. The influence of the new general ophthalmic services (GOS) contract in optometrist referrals for glaucoma in Scotland. *Eye (Lond)* 2009; **23**(2): 351–355.

2. Ang MH, Baskaran M, Kumar RS, et al. National survey of ophthalmologists in Singapore for the assessment and management of asymptomatic angle closure. *J Glaucoma* 2008; **17**(1): 1–4.

3. Azuara-Blanco A, Burr J, Thomas R, Maclennan G & McPherson S. The accuracy of accredited glaucoma optometrists in the diagnosis and treatment recommendation for glaucoma. *Br J Ophthalmol* 2007; **91**(12): 1639–1643.

4. Banes MJ, Culham LE, Bunce C, Xing W, Viswanathan A & Garway-Heath D. Agreement between optometrists and ophthalmologists on clinical management decisions for patients with glaucoma. *Br J Ophthalmol* 2006; **90**(5): 579–585.

5. Chawla A, Patel I, Yuen C & Fenerty C. Patterns of adherence to NICE glaucoma guidance in two different service delivery models. *Eye* 2012; **26**(11): 1412–1417.

6. Cheng J, Beltran-Agullo L, Trope GE & Buys YM. Assessment of the quality of glaucoma referral letters based on a survey of glaucoma specialists and a glaucoma guideline. *Ophthalmology* 2014; **121**(1): 126–133.

7. Coleman AL, Yu F & Evans SJ. Use of gonioscopy in Medicare beneficiaries before glaucoma surgery. *Journal of Glaucoma* 2006; **15**(6): 486–493.

8. Elam AR, Blachley TS & Stein JD. Geographic Variation in the Use of Diagnostic Testing of Patients with Newly Diagnosed Open-Angle Glaucoma. *Ophthalmology* 2016; **123**(3): 522–531.

9. El-Assal K, Foulds J, Dobson S & Sanders R. A comparative study of glaucoma referrals in Southeast Scotland: effect of the new general ophthalmic service contract, Eyecare integration pilot programme and NICE guidelines. *BMC ophthalmol* 2015; **15**: 172.

10. Fung SS, Lemer C, Russell RA, Malik R & Crabb DP. Are practical recommendations practiced? A national multi-centre cross-sectional study on frequency of visual field testing in glaucoma. *Br J Ophthalmol* 2013; **97**(7): 843–847.

11. Ho S & Vernon SA. Decision making in chronic glaucoma–optometrists vs ophthalmologists in a shared care service. *Ophthalmic Physiol Opt* 2011; **31**(2): 168–173.

12. Khan S, Clarke J & Kotecha A. Comparison of optometrist glaucoma referrals against published guidelines. *Ophthalmic Physiol Opt* 2012; **32**(6): 472–477.

13. Liu L. Australia and New Zealand survey of glaucoma practice patterns. *Clin Experiment Ophthalmol* 2008; **36**(1): 19–25.

14. Lockwood AJ, Kirwan JF & Ashleigh Z. Optometrists referrals for glaucoma assessment: a prospective survey of clinical data and outcomes. *Eye* 2010; **24**(9): 1515–1519.

15. Marks JR, Harding AK, Harper RA, et al. Agreement between specially trained and accredited optometrists and glaucoma specialist consultant ophthalmologists in their management of glaucoma patients. *Eye* 2012; **26**(6): 853–861.

16. Ong SS, Sanka K, Mettu PS, et al. Resident compliance with the american academy of ophthalmology preferred practice pattern guidelines for primary open-angle glaucoma. *Ophthalmology* 2013; **120**(12): 2462–2469.

17. Patel UD, Murdoch IE & Theodossiades J. Glaucoma detection in the community: does ongoing training of optometrists have a lasting effect? *Eye* 2006; **20**(5): 591–594.

18. Quigley HA, Friedman DS & Hahn SR. Evaluation of practice patterns for the care of open-angle glaucoma compared with claims data: the Glaucoma Adherence and Persistency Study. *Ophthalmology* 2007; **114**(9): 1599–1606.

19. Scully ND, Chu L, Siriwardena D, Wormald R & Kotecha A. The quality of optometrists' referral letters for glaucoma. *Ophthalmic Physiol Opt* 2009; **29**(1): 26–31.

20. Shah S & Murdoch IE. NICE – impact on glaucoma case detection. *Ophthalmic Physiol Opt* 2011; **31**(4): 339–342.

21. Solano-Moncada F, Dymerska M, Jefferys JL & Quigley HA. Adherence With the Use of Target Intraocular Pressure for Glaucoma Patients in a Large University Practice. *J Glaucoma* 2016; **25**(4): e424–432.

22. Stead R, Azuara-Blanco A & King AJ. Attitudes of consultant ophthalmologists in the UK to initial management of glaucoma patients presenting with severe visual field loss: a national survey. *Clin Experiment Ophthalmol* 2011; **39**(9): 858–864.

23. Swamy L, Smith S & Radcliffe NM. Optic nerve complex imaging in glaucoma Medicare beneficiaries. *Ophthalmic Epidemiol* 2012; **19**(4): 249–255.

24. Syam P, Rughani K, Vardy SJ, et al. The Peterborough scheme for community specialist optometrists in glaucoma: a feasibility study. *Eye* 2010; **24**(7): 1156–1164.

25. Theodossiades J, Myint J, Murdoch IE, Edgar DF & Lawrenson JG. Does optometrists' self-reported practice in glaucoma detection predict actual practice as determined by standardised patients? *Ophthalmic Physiol Opt* 2012; **32**(3): 234–241.

26. Vorwerk C, Thelen U, Buchholz P & Kimmich F. Treatment of glaucoma patients with insufficient intraocular pressure control: a survey of German ophthalmologists in private practice. *Curr Med Res Opin* 2008; **24**(5): 1295–1301.

27. Zangerl B, Hayen A, Mitchell P, Jamous KF, Stapleton F & Kalloniatis M. Therapeutic endorsement enhances compliance with national glaucoma guidelines in Australian and New Zealand optometrists. *Ophthalmic Physiol Opt* 2015; **35**(2): 212–224.

28. Zebardast N, Solus JF, Quigley HA, Srikumaran D & Ramulu PY. Comparison of resident and glaucoma faculty practice patterns in the care of open-angle glaucoma. *BMC Ophthalmol* 2015; **15**: 41.

29. Al-Ubaidi BA, Al-Khadraji MA & Al-Hermi B. Measuring adherence rate to quality indicators for diabetes care identified by primary health care in Bahrain. *Saudi Medical Journal* 2014; **35**(9): 975–980.

30. Burgmann K, Fatio S, Jordi B & Rutishauser J. Medical care of type 2 diabetes mellitus in light of international and national recommendations: a retrospective analysis. *Swiss Medical Weekly* 2013; **143**: w13871.

31. Chou CF, Zhang X, Crews JE, Barker LE, Lee PP & Saaddine JB. Impact of geographic density of eye care professionals on eye care among adults with diabetes. *Ophthalmic Epidemiology* 2012; **19**(6): 340–349.

32. Hutchins E, Coppell KJ, Morris A & Sanderson G. Diabetic retinopathy screening in New Zealand requires improvement: results from a multi-centre audit. *Aust N Z J Public Health* 2012; **36**(3): 257–262.

33. Mc Hugh S, Marsden P, Brennan C, et al. Counting on commitment; the quality of primary care-led diabetes management in a system with minimal incentives. *BMC health services research* 2011; **11**: 348.

34. Preti RC, Saraiva F, Junior JA, Takahashi WY & da Silva ME. How much information do medical practitioners and endocrinologists have about diabetic retinopathy? *Clinics (Sao Paulo, Brazil)* 2007; **62**(3): 273–278.

35. Slater J & Chakman J. Referral of diabetic macular oedema by Australian optometrists: comment. *Clin Experiment Ophthalmol* 2011; **39**(9): 923–924.

36. Ting DS, Ng JQ, Morlet N, et al. Diabetic retinopathy management by Australian optometrists. *Clin Experiment Ophthalmol* 2011; **39**(3): 230–235.

37. Tseng VL, Greenberg PB, Scott IU & Anderson KL. Compliance with the American Academy of Ophthalmology Preferred Practice Pattern for Diabetic Retinopathy in a resident ophthalmology clinic. *Retina* 2010; **30**(5): 787–794.

38. Wong MCS, Wang HHX, Kwan MWM, et al. The adoption of the Reference Framework for diabetes care among primary care physicians in primary care settings: A cross-sectional study. *Medicine (United States)* 2016; **95 (31) (no pagination)**(e4108).

39. Yuen J, Clark A, Ng JQ, et al. Further survey of Australian ophthalmologist's diabetic retinopathy management: did practice adhere to National Health and Medical Research Council guidelines? *Clin Experiment Ophthalmol* 2010; **38**(6): 613–619.

40. Charkoudian LD, Gower EW, Solomon SD, Schachat AP, Bressler NM & Bressler SB. Vitamin usage patterns in the prevention of advanced age-related macular degeneration. *Ophthalmology* 2008; **115**(6): 1032–1038 e1034.

41. Lawrenson JG & Evans JR. Advice about diet and smoking for people with or at risk of age-related macular degeneration: a cross-sectional survey of eye care professionals in the UK. *Bmc Public Health* 2013; **13**.

42. Muhammed S, Yuksel H, Sahin A, et al. Approach of Turkish ophthalmologists to micronutrition in age-related macular degeneration. *Arq Bras Oftalmol* 2015; **78**(1): 10–14.

43. Parodi MB, Zucchiatti I, Cicinelli MV, Cascavilla ML & Bandello F. Nutritional supplementation in age-related macular degeneration. *Retina* 2016; **36**(6): 1119–1125.

44. Gomaa A & Liu C. Nd:YAG laser capsulotomy: a survey of UK practice and recommendations. *Eur J Ophthalmol* 2011; **21**(4): 385–390.

45. Lash SC, Prendiville CP, Samson A, Lewis K, Munneke R & Parkin BT. Optometrist referrals for cataract and "Action on Cataracts" guidelines: are optometrists following them and are they effective? *Ophthalmic Physiol Opt* 2006; **26**(5): 464–467.

46. Park JC, Ross AH, Tole DM, Sparrow JM, Penny J & Mundasad MV. Evaluation of a new cataract surgery referral pathway. *Eye* 2009; **23**(2): 309–313.

47. Niemiec ES, Anderson KL, Scott IU & Greenberg PB. Evidence-based management of resident-performed cataract surgery: an investigation of compliance with a preferred practice pattern. *Ophthalmology* 2009; **116**(4): 678–684.

48. Downie LE & Keller PR. The Self-Reported Clinical Practice Behaviors of Australian Optometrists as Related to Smoking, Diet and Nutritional Supplementation. *Plos One* 2015; **10**(4).

49. Shah R, Edgar DF, Rabbetts R, et al. The content of optometric eye examinations for a young myope with headaches. *Ophthalmic Physiol Opt* 2008; **28**(5): 404–421.

50. Shah R, Edgar DF, Spry PG, et al. Glaucoma detection: the content of optometric eye examinations for a presbyopic patient of African racial descent. *Br J Ophthalmol* 2009; **93**(4): 492–496.

51. Shah R, Edgar DF, Harle DE, et al. The content of optometric eye examinations for a presbyopic patient presenting with symptoms of flashing lights. *Ophthalmic Physiol Opt* 2009; **29**(2): 105–126.

52. Lin IC, Gupta PK, Boehlke CS & Lee PP. Documentation of conformance to Preferred Practice Patterns in caring for patients With dry eye. *Arch Ophthalmol* 2010; **128**(5): 619–623.

53. Downie LE, Keller PR & Vingrys AJ. An Evidence-Based Analysis of Australian Optometrists' Dry Eye Practices. *Optometry and Vision Science* 2013; **90**(12): 1385–1395.

54. Hau S, Ehrlich D, Binstead K & Verma S. An evaluation of optometrists' ability to correctly identify and manage patients with ocular disease in the accident and emergency department of an eye hospital. *Br J Ophthalmol* 2007; **91**(4): 437–440.

55. Jin YP, Chow AHY, Colpa L & Wong AMF. Clinical translation of recommendations from randomized clinical trials on patching regimen for amblyopia. *Ophthalmology* 2013; **120**(4): 657–662.

56. Gupta PK, Freedman SF & Lee PP. Conformance with preferred practice patterns in caring for children with esotropia. *J Pediatr Ophthalmol Strabismus* 2010; **47**(3): 145–149; quiz 150–141.

57. Nguyen QD, Hatef E, Kayen B, et al. A cross-sectional study of the current treatment patterns in noninfectious uveitis among specialists in the United States. *Ophthalmology* 2011; **118**(1): 184–190.
